# Sol-Gel Functionalized Polyurethane Foam-Packed Mini-Column as an Efficient Solid Extractor for the Rapid and Ultra-Trace Detection of Textile Dyes in Water

**DOI:** 10.3390/gels9110884

**Published:** 2023-11-08

**Authors:** Mohammed A. Ghandourah, Mohammad I. Orif, Radwan K. Al-Farawati, Mohammad S. El-Shahawi, Ramadan H. Abu-Zied

**Affiliations:** 1 Department of Marine Chemistry, Faculty of Marine Sciences, King Abdulaziz University, P.O. Box 80207, Jeddah 21589, Saudi Arabia; mioraif@kau.edu.sa (M.I.O.); rfarawati@kau.edu.sa (R.K.A.-F.); rabuzied@kau.edu.sa (R.H.A.-Z.); 2 Department of Chemistry, Faculty of Sciences, King Abdulaziz University, P.O. Box 80207, Jeddah 21589, Saudi Arabia; malsaeed@kau.edu.sa

**Keywords:** mini-column, sol-gel functionalized polyurethane foams solid extractor, breakthrough capacity curve, spectrophotometric determination, wastewater, reusability

## Abstract

Textile dyes widely used in industrial products are known as a major threat to human health and water ecological security. On the other hand, sol gel represents a principal driver of the adoption of dispersive solid-phase microextractors (d-µ SPME) for pollutants residues in water. Thus, the current study reports a new and highly rapid and highly efficient hybrid sol-gel-based sponge polyurethane foam as a dispersive solid-phase microextractor (d-µ-SPME) platform packed mini-column for complete preconcentration and subsequent spectrophotometric detection of eosin Y textile dye in wastewater. The unique porous structure of the prepared sol-gel immobilized polyurethane foams (sol-gel/PUF) has suggested its use for the complete removal of eosin Y dye (EY) from water. In the mini-column, the number (N) of plates, the height equivalent to the theoretical plates (HETP), the critical capacity (CC), and the breakthrough capacities (BC) of the hybrid sol-gel-treated polyurethane foams towards EY dye were determined via the breakthrough capacity curve at various flow rates. Under the optimum condition using the matrix match strategy, the linear range of 0.01–5 µg L^−1^, LODs and LOQs in the range of 0.006 µg L^−1^, and 0.01 µg L^−1^ for wastewater were achieved. The intra-day and inter-day precisions were evaluated at two different concentration levels (0.05 and 5 μg L^−1^ of dye) on the same day and five distinct days, respectively. The analytical utility of the absorbents packed in pulses and mini-columns to extract and recover EY dye was attained by 98.94%. The column could efficiently remove different dyes from real industrial effluents, and hence the sol-gel/PUF is a good competitor for commercial applications. The findings of this research work have strong potential in the future to be used in selecting the most suitable lightweight growing medium for a green roof based on stakeholder requirements. Therefore, this study has provided a convenient pathway for the preparation of compressible and reusable sponge materials from renewable biomass for efficient removal of EY from the water environment.

## 1. Introduction

In today’s era, freshwater demand has increased significantly, as it is regarded as an essential component of the earth’s ecosystem and plays an important role in modern life [1,2]. Freshwater resources like lakes, rivers, and underground water face a lot of challenges, as scarcity is becoming a big issue around the world. Thus, one of the challenges of the twenty-first century is related to the discharge and disposal of industrial effluents and wastewater resulting from the textile industry [3,4]. Textile dyes, even at micro- and ultra-micro levels, are among the most hazardous and highly harmful contaminants in the aquatic environment [5,6]. A variety of textile dyes are the main sources of surface and groundwater pollution [6,7]. All proposed methods, such as ultrafiltration membrane [8], ion exchange [9], electrocoagulation [10], advanced oxidation process [11], photocatalytic degradation [12], adsorption [13], coagulation and flocculation [14], and phytoremediation [15] have their own drawbacks that make their use challenging. Thus, the development of fast and reliable solid-phase extractors is of critical need for the rapid removal of textile dyes by low-cost solid-phase extractors from aquatic environments, including wastewater, which has become a major global challenge for environmental protection [16]. One of the new methods used for wastewater treatment is the use of dispersive, low-cost solid adsorbent, which has attracted the attention of researchers in recent years due to its advantages such as simplicity, environmental friendliness, and low cost [16,17].

Recently, eosin yellow (EY) textile dye has received great awareness because of its high solubility in water and its distinctive fluorescence [8]. Eosin yellow dye may cause severe skin and eye irritation [18,19]. Direct contact of eosin yellow dye with the eye can also be a source of eternal damage to the cornea by destroying retinal ganglion cells placed near the retina’s inner surface [20,21]. Thus, great consideration has been oriented towards the minimization and/or complete removal of textile dyes from aquatic environmental waste water [22,23].

In today’s era, according to the importance of water treatment, great attempts have been made in the adsorption removal process via the synthesis and functionalization of various adsorbents to improve their adsorption behavior [24]. However, their liquidness or solubility restricts their utility, specifically in the adsorptive removal processes, but their immobilization on solid supports was found to be an effective way to overcome these drawbacks [25]. Thus, numerous solid-phase extractors, such as the use of eucalyptus tree leaf biomass [25], modified xanthan gum/silica hybrid nanocomposite adsorbent [26], immobilized TiO_2_/chitosan-montmorillonite [27,28], non-conventional, low-cost adsorbents [29,30], remediation of RhB Organic Dye Using α-MnO_2_ under visible-light irradiation [31], and sol-gel sorbents in sorptive microextraction [32]. Polyurethane foams (PUFs) [33,34,35,36] as sponges, as chemically stable, available, low-cost, and easy solid-phase extractors to reuse for dyes and other organic pollutants removal [33], have been established for the treatment of wastewater to solve the major worldwide crisis of textile dyes from water [34,35]. Sol-gel technology has already aided in the development of a significant variety of innovative solid sorbents with enormous surface areas, good thermal and solvent stability, and high selectivity [32]. Solid-phase microextraction based on the sol-gel technique offers a rapid, easy, and suitable pathway to prepare chemically bonded and stable SPME coatings [32]. To the best of our knowledge, no studies on the use of sol-gel-modified PUFs as solid-phase microextractors are known to date. Thus, the overall goals of the current study are focused on: (i) studying the impact of various parameters that facilitates EY dye retention from the aqueous media by the sol-gel modified PUFS packed column; (ii) developing a low cost spectrophotometric method for trace and ultra-trace levels of selected textile dye EY; and finally, (iii) testing the re-usability of the established sol-gel dispersed PUFs extractor. The current strategy will contribute effectively to further progress and refinement in the removal and/or minimization of textile dye residues from the aquatic environment, such as industrial wastewater and underground water samples. A cohesive collaboration of industry and academic institutes will be progressively anticipated to put sol-gel/PUFs platforms into market distribution and mass fabrication.

## 2. Results and Discussion

### 2.1. Preliminary Study

**Figure 1:**
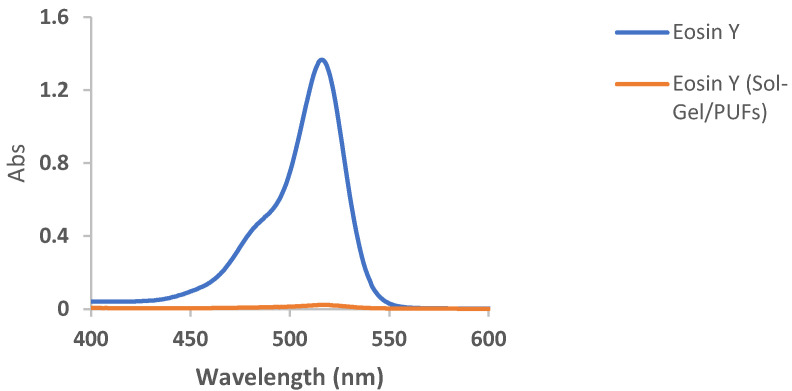
Electronic spectra of EY dye in an aqueous solution of pH < 3 before and after extraction into sol-gel-treated PUFs after 60 min of shaking time.

Selection and/or improvement of an effective and low-cost dispersive solid-phase microextractor (d-µ SPME) are vital in water treatment [37,38,39]. The interaction of the tested analyte in the aquatic environmental samples with the developed solid extractor and its available active sites is also of great concern. Thus, the establishment of an extremely efficient, high-sorption capacity, and high enrichment factor dispersive solid-phase microextractor sorbent for complete removal of the target textile dye from the aqueous phase is of prime importance [39]. PUFs have great permeability, convection dominated mass transfer, operation at high linear velocity, a unique membrane-like structure, resilience properties, ease of modification, and chemical stability at a wide range of pH compared to other conventional sorbents [36]. Thus, the selection of nanomaterials with exceptional properties, e.g., a great surface area and a small particle size, such as sol-gel for PUF modifications also makes PUFs an ideal solid platform for the complete removal of the potent target species [40]. Initial study on the EY dye retention from the aqueous solution of pH < 3 by the developed sol-gel functionalized PUFs sorbent revealed complete uptake of the EY dye, as demonstrated in Figure 1. Thus, in the sequence study, characterization and the analytical utility of the sol-gel-treated PUFs as d-µ SPME platform-packed mini-columns for removal and/or determination of EY dye are discussed below.

### 2.2. Characterization of Sol-Gel

**Figure 2:**
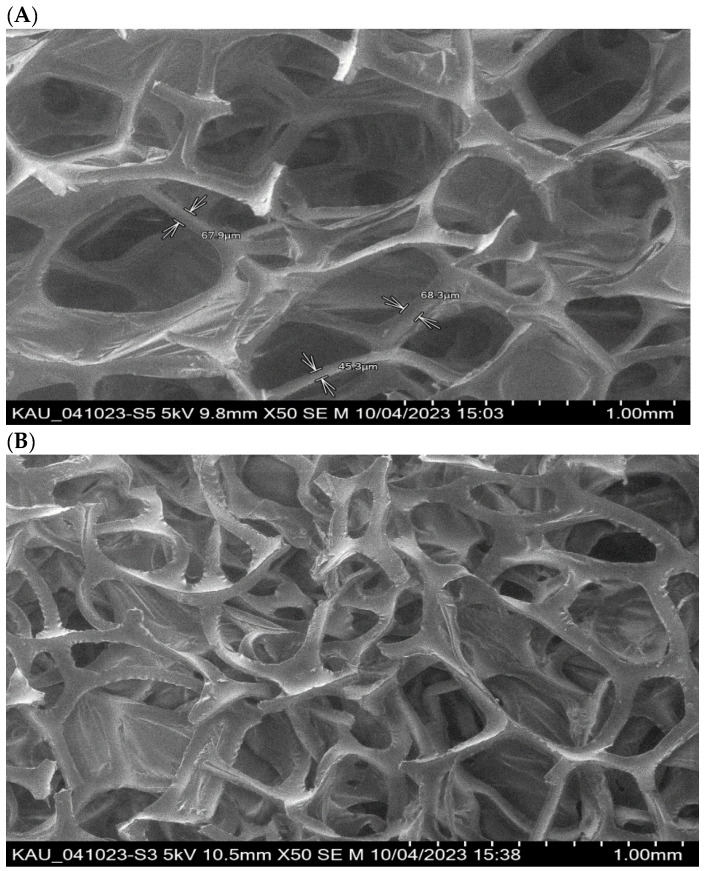
Scanning electron micrographs of PUFs (**A**) and sol-gel-treated PUFs (**B**) structure. **Top**—9.8 × 50; **Bottom**—10.5 × 50.

The SEM images of the PUFs and sol-gel-treated PUFs are shown in Figure 2A,B. The membrane-like structure of the flexible PUFs with and without sol-gel is quite clear, in agreement with the data reported earlier [35,36]. The polyhedral structure is clearly visible, and the polyhedrals, on average, are quasi-spherical pentagonal dodecahedra, in agreement with the data reported [36]. On the other hand, the polymer and the sol-gel are distributed between the walls of the bubbles and the lines where bubbles intersect. The sol-gel-immobilized PUFs were supported by recording the EDX spectra of the PUF-treated sol-gel. The spectrum revealed peaks at 0.09, 0.25, 0.5, and 1.75 keV for C, N, O, and Si, respectively.

The functionality of the sol-gel, its morphology, including its particle size, and its surface area may play a vital role in the accessibility of the tested textile dyes from the test aqueous solution. Thus, DLS analysis after 30 days of the preparation of the sol-gel material was recorded. The DLS analysis revealed the appearance of one band that lays within the range 2–50 nm and is centered between 5 and 20 nm, indicating that the network is composed of a distribution of particles with the most ranging between 5 and 20 nm [41,42]. Thus, the developed sol-gel material can be used as a nanomaterial that can be immobilized onto PUF sorbent.

### 2.3. Chromatographic Separation

**Figure 3:**
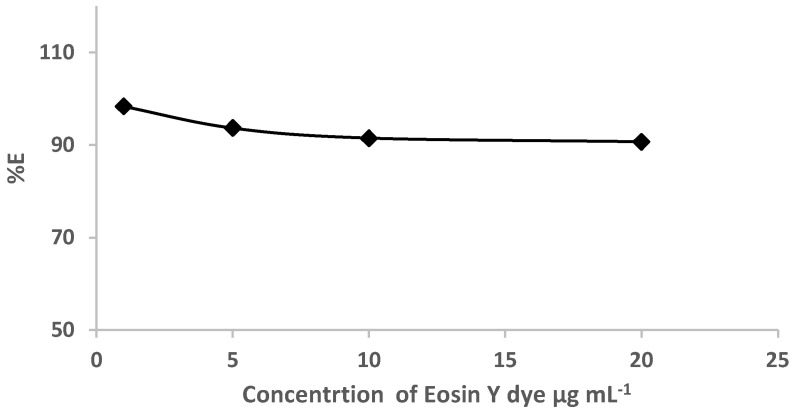
Plot of % E versus eosin dye concentrations (1.0 to 20.0 μg mL^−1^) percolated through the sol-gel/PUFs packed mini-column at flow rate of 10 ± 2 mL min.^−1^.

Based on the retention profile of EY (Figure 1) from water by the sol-gel-treated PUFs, great attempts were performed to use sol-gel-treated PUF-packed column (flow mode) for the removal of eosin dye. Preliminary experiments involving the use of the sol-gel-treated PUFs sorbent (0.3 ± 0.002 g) packed in a mini-column for the retention of various known concentrations (1.0–20 μg mL^−1^) of EY dye in deionized water (100 mL) at pH ≤ 3 were individually passed through the mini-column at 10 ± 2 mL min^−1^. A blank experiment was also performed at the same flow rate. Spectrophotometric measurement of the dye in the effluent was performed. The results are illustrated in Figure 3. The data revealed complete retention of the dye by the used extractor.

**Figure 4:**
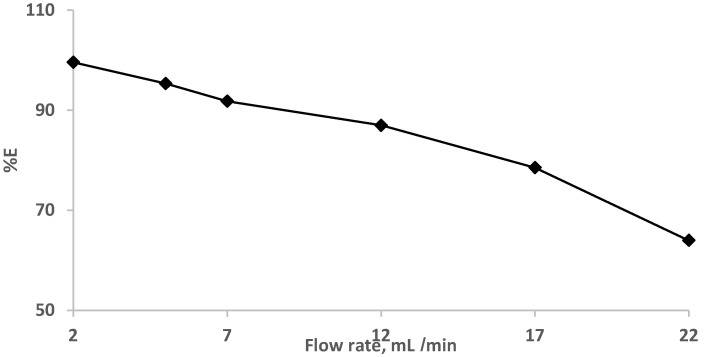
Impact of flow rate on eosin Y dye (10 μg mL^−1^) retention onto sol-gel/PUFs packed mini-column at various flow rates.

The impact of the flow rates (2.0–22 mL min^−1^) was also studied at a constant EY concentration (10 µg/mL^−1^). The results are demonstrated in Figure 4, where complete retention (90.0–100%) of the dye was achieved at flow rates less than 7.0 mL min^−1^. and decreased at high flow rates.

**Figure 5:**
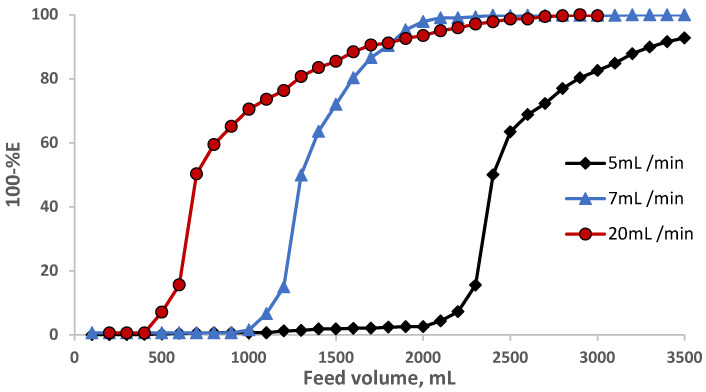
Breakthrough capacity curve for eosin Y retention on the PUF-packed mini-columns at various flow rates.

The performance of the sol-gel foam-packed mini-column towards eosin Y retention was determined from the calculation of the number (N) and the height equivalent to the theoretical plate (HETP), the critical capacity (CC), and the breakthrough capacity (BC). Thus, in a separate experiment, an aqueous solution (3.5 L) containing EY dye at a known concentration (10 g mL^−1^) was percolated through the sol-gel-treated PUFs (0.5 ± 0.04 g) packed mini-column at various flow rates (5, 10, and 20 mL min^−1^) under the optimal experimental conditions. The results are shown in Figure 5. The N and HETP values were calculated employing the following equation [33,34]:

$$N=\frac{V_{50}V^{\prime }}{{\left(V_{50}V^{\prime }\right)}^{2}}=\frac{L}{\mathrm{HETP}}$$

where V_50_ = the volume of the feed solution at the center (50% extraction) of the *S*-shaped curve, i.e., at 50% extraction, and V′ is the volume at which the feed solution has a value of 0.1578 of the initial concentration, i.e., at 15.78% non-extraction of the tested compound. The computed values of N were found to be 552, 156, and 30, and the HETP was equal to 3.4 × 10^−3^, 0.01, and 0.05 mm at a 5, 10, and 20 mL min^−1^ flow rate, respectively. The HETP values increased (the column efficiency decreased) with increasing the flow rate from 5 to 20 mL min^−1^. The breakthrough capacity (BC) was also calculated using the following equation [33,34]:

$$\mathrm{BC}=\frac{V_{50}\times C_{0}}{W}$$

The CC, i.e., the amount of analyte retained on the column before first detection in the effluent per one gram of the solid sorbent, was further calculated. The values of the CC for eosin Y were found to be 48, 24, and 10 mg g^−1^ sorbent, respectively, confirming the good performance of sol-gel PUF-packed mini-columns in enrichment and recovery of the tested EY dye.

**Figure 6:**
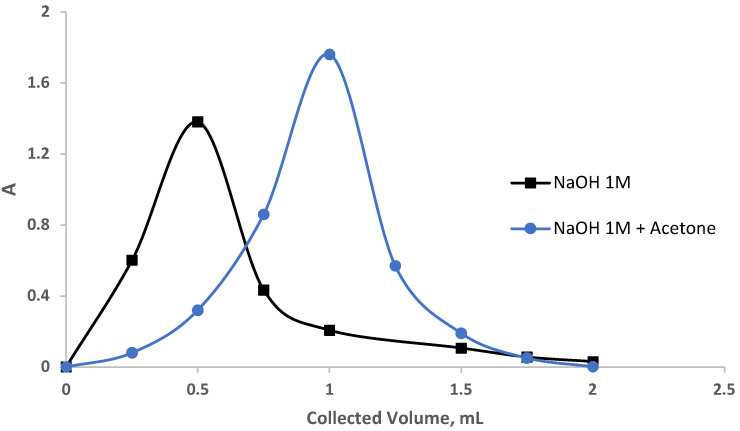
Recovery of the dye eosin yellow from the sol-gel/PUFs column.

The performance of the sol-gel-treated PUFs packed in a mini-column was further calculated by the chromatogram (Gluenkauf) method [34] as follows: An aqueous solution (100 mL) spiked with EY (5.0 μg mL^−1^) individually was percolated through sol-gel-immobilized PUF-packed columns at a 7 mL min^−1^ flow rate under the optimized parameters of dye retention. Complete retention of the EY dye was achieved, as detected by measuring the dye in the effluent solution. Attempts to recover the dye from the sorbent-packed column were performed using a variety of eluating agents, e.g., NaOH (5.0 mL, 1.0 mol L^−1^), acetone, and acetone-NaOH (1.0 mol L^−1^) at 1:1 ratio (*v*/*v*) individually at a 0.25 mL/min flow rate. Various fractions (250 μL) of the eluating agent at a 0.25 mL min^−1^ flow rate were percolated through the sorbent-packed column. The absorbance of the eluate fractions was determined at the optimized wavelength against a reagent blank. The obtainable results are demonstrated in Figure 6. The values of N and HETP were then calculated employing the following equation:

$$N=\frac{8V_{\max}^{2}}{W^{2}}=\frac{L}{\mathrm{HETP}}$$

where V_max_ is the volume of eluate at peak maximum, W is the peak at 1/e times the maximum solute concentration, and L is the length of the foam bed in mm, respectively. The values of N and HETP using NaOH and acetone—NaOH as follows were found to be 13.4, 7.9, and 0.13, 0.25, respectively. These results are much better than the results reported earlier for the chemical modification of chitosan by tetraethylenepentamine [42] and using ethylenediamine-modified chitosan [43].

Based on the values of N, HETP, BC, and CC, the analytical utility of sol-gel/PUFs sorbent-packed mini-column was successfully tested for complete collection, recovery, and spectrophotometric determination of EY dye at trace and ultra-trace levels (0.001–1.0 µg mL^−1^) in deionized water. For this purpose, aqueous solutions (100 mL) spiked with various known concentrations of EY were percolated individually through sol-gel/PUF-packed mini-columns at a 7 mL min^−1^ flow rate versus a blank experiment. Complete extraction of EY from the test solutions was achieved, as indicated by the absorbance of the effluent. The retained species were successfully recovered with NaOH (4M) at 0.25 mL min^−1^, and the results are given in Table 1. A complete recovery (100–104 ± 0.4) of dye was achieved. These results support the analytical utility of the established sorbent packed for complete removal and quantification of the dye from environmental water samples.

### 2.4. Analytical Applications

The analytical utility of the established sol-gel-treated PUFs was tested for the extraction, recovery, and subsequent determination of EY dye in tap and red seawater samples following the recommended procedures. Typical results are given in Table 2 and Table 3. A reasonable recovery percentage in the range of 92.04 ± 0.2 to 106.02 ± 1.02 was successfully achieved, confirming the applicability of the established extractor for complete dye removal from environmental water samples.

### 2.5. Reusability of the Established Solid Platform

Under the optimized analytical parameters of dye (eosin and/or Congo red) sorption and recovery, the reusability of the established solid-phase platform was tested as follows: After each column experiment, the SPE was collected, washed several times with deionized water and acetone, dried at room temperature between filter paper, and repacked in the column for the extraction process. Over 94 ± 5.4% of EY dye sorption and recovery were achieved after 3–4 times without significant decrease (±4%) in analyte uptake and stripping by the established solid phase microextractor.

## 3. Conclusions

The established extractor is simple and cost-effective to use for the removal of textile dyes from water. The synthesis of novel hybrid sol-gel sorbent-treated solid-phase extractors as nanocomposites via incorporating a diverse range of nanomaterials and various inorganic precursors in the sol-gel preparation looks promising in SPME. The developed extractor will be used for monitoring ultra-trace levels of the dye in various environmental samples. The distinctive porous arrangement of the sol-gel immobilized polyurethane foams (sol-gel/PUF), indicating its potential, has been proposed for effectively eliminating eosin Y dye (EY) from water. The established sol-gel-treated PUF solid platform will serve as a routine approach for the complete removal and subsequent determination of various organic and inorganic pollutants in environmental water samples. The dependent claims outline the beneficial forms of hybrid sol-gel treatments applied to polyurethane surfaces. They also examine how specific analytical factors govern the uptake of textile dyes as well as the methods for extracting organic pollutants (both in flow and pulse modes). The invention highlights a preferred embodiment involving a robust and effective nanoparticle-infused hybrid sol-gel treatment, achieved by firmly attaching a silane compound to polyurethane foams through irreversible bonding. The study’s innovative extraction method for textile dye detection has implications for environmental assessment, analytical chemistry, and water quality monitoring. Its applications extend to environmental monitoring, water treatment, quality control, and research and development. Recommendations for the future include further validation, method optimization, and interdisciplinary collaboration to address the pressing issue of textile dye pollution in water.

## 4. Materials and Methods

### 4.1. Materials and Reagents

Analytical reagent-grade chemicals were used as received. All glassware was washed with conc. HNO_3_, rinsed with deionized water prior to use, and dried at 80 °C in an oven. All aqueous solutions were made up of deionized water. A standard stock solution (1000 µg mL^−1^) of EY dye was prepared by dissolving the required weight of EY dye in deionized water (1000 mL). More diluted solutions (0.001–20 µg mL^−1^) of EY dye were prepared by appropriate dilution of the stock solution of the dye in deionized water. The compounds NaOH and/or acetone were used as eluting agents in min-column (flow) and pulse procedures. White sheets of polyether-type-based open-cell polyurethane foams (PUFs) were purchased from the local market in Jeddah City, Saudi Arabia. The foam cubes of almost 1–1.5 cm^3^ were cut from foam sheet and were washed with hydrochloric acid (10% *v*/*v*), deionized water until the washing solutions were free from chloride ions [43], then washed with acetone to remove organic contaminants, and lastly dried at 80 °C for 3 h in an oven.

### 4.2. Instrumentation

**Figure 7:**
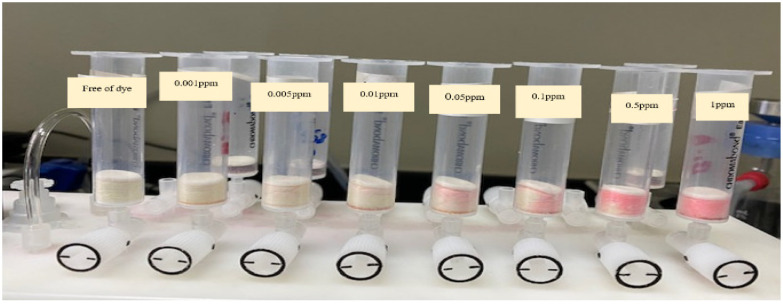
Separation of eosin Y dye (0.001–1 g mL^−1^) on a solid-phase extraction manifold (Agilent Technologies).

Chromatographic extraction was carried out on a Solid-Phase Extraction Manifold (Agilent Technologies, Santa Clara, CA, USA). A UV-Vis-spectrophotometer (Shimadzu UV-Vis 1800, Shimadzu, Columbia, MD, USA) (190–1100 nm) with 10.0 and 1.0 mm (path width) quartz cells was used for recording the electronic spectra and absorbance reading of the dye before and after extraction at the optimized wavelength. A series of digital micropipettes (Volac) (Model 3505, Unit 2, Vernon Drive, Battlefield Enterprise Park, Shrewsbury, UK) (100-1000, 20–200, and 0.5–10 μL). A scanning electron microscopy (SEM) (JEOL-JSM6301-F) (Peabody, MA, USA) was used for characterization of the surface topography and roughness of the PUFs and sol-gel-modified PUFs. A series of mini-column-packed sol-gel-treated polyurethane foam-packed columns of different capacities (10, 20, 50, and 100 mL) was used (Figure 7). A Milli-Q Plus system from Millipore, Bedford, MA, USA, was used for providing ultra-pure water. The synthesized sol-gel particle sizes were measured using a Malvern Nano-ZS Dynamic Light Scattering (DLS) instrument. The sol-gel was first filtered through a 0.45 μm Whatman syringe filter and diluted with isopropyl alcohol in a 1:10 (*v*/*v*) dilution prior to DLS analysis to prevent aggregation of the particles and to enable the characterization of the individual particles.

### 4.3. Preparation of Sol-Gel Materials

The sol-gel matrix was prepared via hydrolysis and condensation reactions of the silicon precursor 3-methacryloxypropyltrimethoxy–silane (H_2_C=C(CH_3_)CO_2_(CH_2_)_3_Si(OCH_3_)_3_] abbreviated as MAPTMS (99% in methanol, Aldrich) and the modified organic zirconium complex, prepared from the chelation of Zr^4^+-n-propoxide (ZPO, Assay 70% in propanol, Sigma Aldrich, Irl., St. Louis, MI, USA). ZPO was 80:20, and the theoretical hydrolysis degree was 50% against the total content of reactive alkoxide groups. The synthesis required a three-step process, as shown in the Electronic Supplementary Information’s (Figure S1), as follows: (i) pre-hydrolysis of the MAPTMS and complexation of ZPO with the chelating agent; (ii) addition of the pre-hydrolyzed alkoxysilane within the zirconate complex; and finally, (iii) hydrolysis of the solution mixture where the MAPTMS is pre-hydrolyzed using an aqueous HNO_3_ (0.1 M) solution with a 1:0.25 ratio. The hydrolysis is performed in a heterogeneous way within 5 min until methanol production becomes sufficient to enable the miscibility of all species in solution. In parallel, ZPO is chelated with MAAH to block two of its alkoxide groups and minimize precipitation when it is in contact with water. These two reactions were achieved simultaneously and allowed to stir for 45 min, and the partially hydrolyzed MAPTMS was added gradually by drop-wise addition to the Zr (VI)-complex. Nutral hydrolysis was then performed after 5 min with deionized water by drop-wise addition to the mixture. A diagram describing the Sol gel preparation is demonstrated in the Electronic Supplementary Information’s (Figure S1).

### 4.4. Synthesis of Sol-Gel Functionalized Polyurethane Foams (d-µ SPME)

An accurate mass (1.0 g) of the PUF cubes was dried and shaken with Sol-gel dissolved in isopropanol (4% *v*/*v*) with constant stirring for 30 min. After shaking, the sol-gel-impregnated PUF cubes were then separated out and dried between sheets of filter paper, as mentioned earlier [44,45]. 

### 4.5. Recommended Procedures

#### 4.5.1. Preparation of the Min-Columns

A cylindrical polyethylene tube with a length of 8.0 cm and a 51.0 mm internal diameter served as a mini-column. The tube was manually packed with the Sol-gel/PUF under suction by fixing the end of the tube to the suction port of the SPE manifold. After effective packing, the suction was continued for 30 min to reduce channeling inside the mini-column. After completion of the task, the mini-columns were washed with 3% (*v*/*v*) ethanol, 0.5 M NaOH, and deionized water, respectively, at a 3.0 mL flow rate.

#### 4.5.2. Mini-Column Assays

In the univariate fixed-bed experiments of mini-column, the parameters assessed were the impact of flow rate and initial dye concentration on adsorption. The EY dye concentrations were 1, 5, 10, and 20 mg mL^−1^. The dye solutions (100.0 mL) were percolated individually through the columns at various flow rates between 2, 5, 7, 12, 17, and 22 mL min^−1^. The retained species were then recovered from the sol-gel-treated PUF packed column with acetone (10 mL) and/or sodium hydroxide (1.0 M), a proper eluating agent, at a flow rate of 1.0–2.0 mL min ^−1^. The absorbances of the recovered solutions were then measured against the reagent blank. The percentage recovery of the dye adsorption or the extracted dye content was estimated by comparing the concentration of the analyte before and after adsorption.

#### 4.5.3. Breakthrough (S-Shaped) Capacity Curve Method

In this experiment, various fractions (200 mL) of the test feed aqueous solution (3.5 L) containing (10 μg mL^−1^) of EY dye at the optimum conditions were passed through the sol-gel/PUF-packed mini-column (0.5g ± 0.002) at 5, 10, and 20 mL min^−1^ flow rates. This was carried out until the concentration of the tested EY dye in the effluent reached that of the feed one. The concentration of the tested EY dye was determined from the absorbance measurement of the effluent at λ_max_ versus reagent blank for each tested EY dye. The critical capacity (CC), the breakthrough capacity (BC), the number (N), and the height equivalent of the theoretical plates (HETP) were finally determined as reported earlier [44,45].

#### 4.5.4. Chromatogram (Gluenkauf Method)

The analytical performance of the sol-gel/PUF-packed (0.3 g ± 0.002) mini-column was also calculated by the chromatogram (Gluenkauf method) as follows: An aqueous solution (100 mL), individually spiked with EY (5.0 μg mL^−1^), was percolated through sol-gel immobilized PUF-packed columns at 7.0 mL min^−1^ flow rate under the optimized parameters of dye retention. Complete retention of the EY dye was achieved, as detected by measuring the dye in the effluent solution. Attempts to recover the dye from the packed were performed using a variety of eluating agents, e.g., NaOH (5.0 mL, 1.0 mol L^−1^), acetone, and acetone-NaOH (1.0 mol L^−1^) at a 1:1 ratio (*v*/*v*) individually at a 0.25 mL min ^−1^ flow rate. Various fractions (250 μL) of the eluating agent at a 0.25 mL min^−1^ flow rate were percolated through the sorbent-packed column. The absorbance of the eluated fractions (eluate) was determined at the optimized wavelength against a reagent blank.

### 4.6. Analytical Applications: Rretention and Recovery of EY Dye by Flow (Mini-Column)

Amounts of 500 mL of tap water, sea water, and industrial water were filtered through a 0.45 µm filter membrane and stored in low-density polyethylene (LDPE) bottles. The procedure was carried out on samples containing EY dye at concentrations in the ranges 0.05–5.0 mg ml^−1^ and 5.0–10.0 μg ml^−1^, respectively, at pH ≤ 3.0, filtered through an adsorbent loaded with PUFs (0.3 g ± 0.002) packed mini-columns. Blank experiments were also performed in the absence of the tested compounds. The retained EY dye was recovered with NaOH (5 mL, 4 M) and acetone (5 mL). The solution was finally adjusted to pH ≤ 3.0 with NaOH, and the absorbance of the total effluent was measured at λ_max_ against the reagent blank.

## Figures and Tables

**Table 1:** Analytical results for the extraction and recovery of EY from deionized water by sol-gel/PUF-packed column at a flow rate of 7 mL min^−1^.

Eosin Y Added, μg/mL	Eosin Y Found, μg/mL	Recovery %
1.0	1.4 ± 0.4	104 ± 0.4
0.5	0.52 ± 0.2	102 ± 0.2
0.1	0.1 ± 0.2	100 ± 4
0.05	0.051 ± 0.04	100 ± 4
0.01	0.01	100 ± 4
0.005	nd.	nd.
0.001	nd.	nd.

nd.—not detected.

**Table 2:** Analytical results of the extraction and recovery of eosin Y dye spiked into tap water samples by the proposed sol-gel/PUF batch mode.

Sample	Eosin Y Dye Added, μg/mL	Eosin Y Dye Found, μg/mL	Recovery, %
Tap water	20	19.86 ± 0.01	99.31 ± 0.1
	10	9.36 ± 0.01	93.65 ± 0.2
	5	4.604 ± 0.01	92.08 ± 0.01

**Table 3:** Results of the extraction and recovery of eosin Y spiked environmental (tap, sea, and waste) water samples by the proposed sol-gel/PUF-packed mini-column.

Sample	Eosin Y Dye Added, μg/mL	Eosin Y Dye Found, μg/mL	Recovery, %
Tap water	5	4.94 ± 0.2	98.94 ± 0.1
	0.5	0.499 ± 0.01	99.8 ± 0.1
	0.1	0.1 ± 0.01	100 ± 0.1
	0.05	0.05 ± 0.01	100 ± 0.01
Sea water	5	5.301 ± 0.021	106.02 ± 1.02
	0.5	0.498 ± 0.01	99.6 ± 0.1
	0.1	0.1 ± 0.01	100 ± 0.1
	0.05	0.05 ± 0.01	100 ± 0.01
Wastewater	5	4.602 ± 0.01	92.04 ± 0.26
	0.5	0.497 ± 0.01	99.4 ± 0.1
	0.1	0.1 ± 0.01	100 ± 0.01
	0.05	0.05 ± 0.01	100 ± 0.01

## Data Availability

The data used to support the findings of this study are included within the article.

## Supplementary Materials

The following supporting information can be downloaded at: https://www.mdpi.com/article/10.3390/gels9110884/s1, Figure S1: A diagram describing the Sol gel preparation.
