# Supplementary material for: Sol-Gel Functionalized Polyurethane Foam-Packed Mini-Column as an Efficient Solid Extractor for the Rapid and Ultra-Trace Detection of Textile Dyes in Water
Source: Gels. 2023 Nov 8;9(11):884. doi: 10.3390/gels9110884 (PMC10670804; doi:10.3390/gels9110884)
Supplement: Supplementary file 1 [file gels-09-00884-s001.zip › gels-2623848-supplementary.pdf]

Sol-gel Functionalized Polyurethane Foams Packed Mini-column as Efficient Solid Extractor for Rapid and Ultra-trace Detection of Textile Dyes in Water

Mohammed A. Ghandourah [1,*], Mohammad I. Orif [1], Radwan K. Al-Farawati [1], Mohammad S. El-Shahawi [2], and Ramadan H. Abu-Zeid [1]

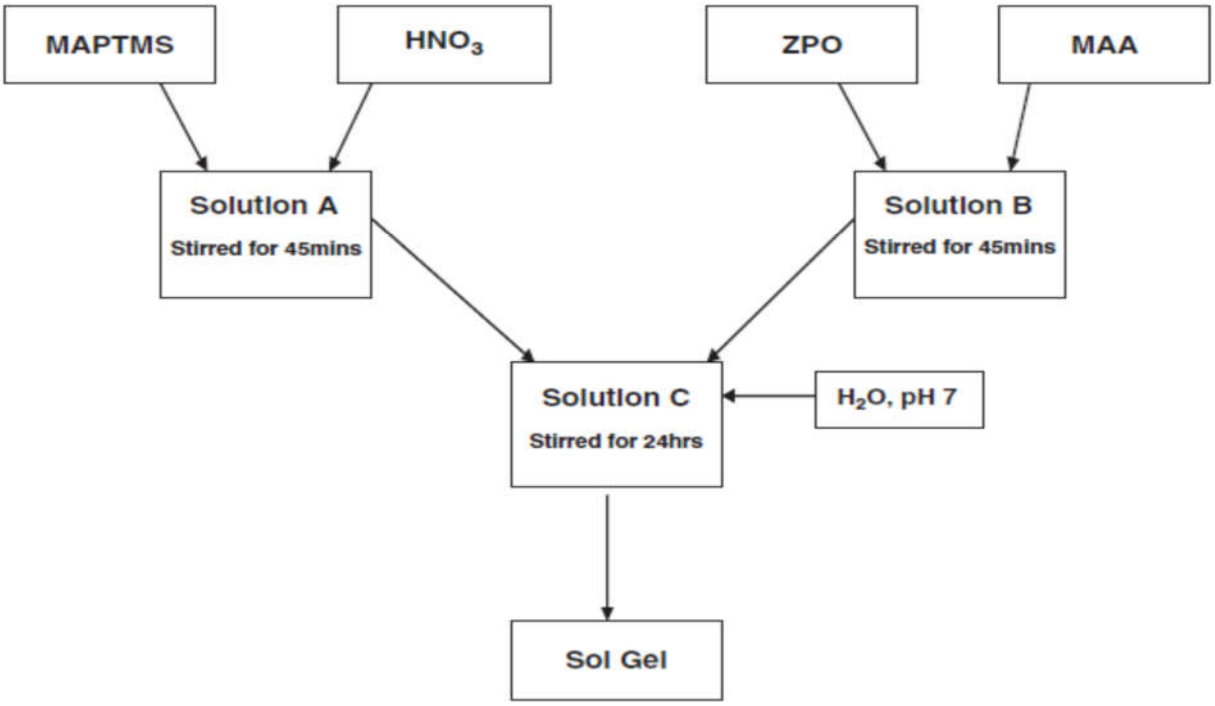

Figure S1. A diagram describing the preparation of the hybrid sol-gel used in the current study.
